# Disrupted Brain Functional Network in Internet Addiction Disorder: A Resting-State Functional Magnetic Resonance Imaging Study

**DOI:** 10.1371/journal.pone.0107306

**Published:** 2014-09-16

**Authors:** Chong-Yaw Wee, Zhimin Zhao, Pew-Thian Yap, Guorong Wu, Feng Shi, True Price, Yasong Du, Jianrong Xu, Yan Zhou, Dinggang Shen

**Affiliations:** 1 Image Display, Enhancement, and Analysis (IDEA) Laboratory, Biomedical Research Imaging Center (BRIC) and Department of Radiology, University of North Carolina at Chapel Hill, Chapel Hill, North Carolina, United States of America; 2 Department of Child & Adolescent Psychiatry, Shanghai Mental Health Center, Shanghai Jiao Tong University, Shanghai, PR China; 3 Department of Radiology, Renji Hospital, Jiao Tong University Medical School, Shanghai Jiao Tong University, Shanghai, PR China; 4 Department of Brain and Cognitive Engineering, Korea University, Seoul, Republic of Korea; Wake Forest School of Medicine, United States of America

## Abstract

Internet addiction disorder (IAD) is increasingly recognized as a mental health disorder, particularly among adolescents. The pathogenesis associated with IAD, however, remains unclear. In this study, we aim to explore the encephalic functional characteristics of IAD adolescents at rest using functional magnetic resonance imaging data. We adopted a graph-theoretic approach to investigate possible disruptions of functional connectivity in terms of network properties including small-worldness, efficiency, and nodal centrality on 17 adolescents with IAD and 16 socio-demographically matched healthy controls. False discovery rate-corrected parametric tests were performed to evaluate the statistical significance of group-level network topological differences. In addition, a correlation analysis was performed to assess the relationships between functional connectivity and clinical measures in the IAD group. Our results demonstrate that there is significant disruption in the functional connectome of IAD patients, particularly between regions located in the frontal, occipital, and parietal lobes. The affected connections are long-range and inter-hemispheric connections. Although significant alterations are observed for regional nodal metrics, there is no difference in global network topology between IAD and healthy groups. In addition, correlation analysis demonstrates that the observed regional abnormalities are correlated with the IAD severity and behavioral clinical assessments. Our findings, which are relatively consistent between anatomically and functionally defined atlases, suggest that IAD causes disruptions of functional connectivity and, importantly, that such disruptions might link to behavioral impairments.

## Introduction

It has been reported that overuse of the internet can lead to altered socio-behavioral characteristics that are similar to those found in substance addictions and pathological gambling [1], [2]. With the soaring number of internet users over the past decades, this problem has been increasingly considered as a serious public health issue [3]. Internet addictions, and computer-related addictions in general, appear to be a wide-spread phenomenon, affecting millions of individuals in the United States and abroad, with the highest rates of incidence occurring among adolescents and college students in developing regions of Asia [3]–[7]. The effect of internet overexposure during young adulthood is of particular clinical and societal significance, as adolescence is a period of significant changes in neurobiology related to decision-making [8] and thereby exhibits a higher susceptibility to affective disorders and addiction [9]–[11]. Since the seminal work by Young [2], internet addiction has attracted significant attention from sociologists, psychologists, psychiatrists, and educators.

The clinical features of behavioral problems related to internet use have been described under various diagnostic criteria, including internet addiction disorder (IAD) [12], pathological internet use [13], and problematic internet use [14]. IAD has been classified as an impulse-control disorder, since it involves maladaptive internet use without any intoxicant, similar to pathological gambling. IAD manifests similar characteristics of other addictions, including the development of academic, financial, and occupational difficulties as a result of addictive behavior and problems in developing and maintaining personal and family relationships. Individuals who are suffering from IAD will spend more time in solitude, which in turn affects their normal social functioning. In the worst cases, patients may experience physical discomfort or medical problems such as carpal tunnel syndrome, dry eyes, backaches, severe headaches, eating irregularities, and disturbed sleep [15], [16]. Moreover, patients are often resistant to treatment of IAD and have a high relapse rate [17], and many of them also suffer from other addictions, such as addiction to drugs, alcohol, gambling, or sex [18].

While IAD is not yet considered as an addiction or mental disorder in the DSM-5 [19], there are ample studies, mainly based on self-reported psychological questionnaires, showing negative consequences in daily life in terms of behavioral components, psychosocial factors, symptom management, psychiatric comorbidity, clinical diagnosis, and treatment outcome [6], [20]–[23]. Besides these behavioral-based analyses, neuroimaging techniques have been applied recently to explore the effect of heavy internet overuse on the structural and functional characteristics of the human brain [7], [24]–[29]. Resting state functional magnetic resonance imaging (R-fMRI), an effective *in vivo* tool for investigating neuronal activities of the brain, has previously been employed to identify possible disruptions of the encephalic functional characteristics in IAD [24], [26], [27], [30]. In [27], regional homogeneity (ReHo) analysis, which measures the consistency of regional low frequency fluctuations (LFF) within brain networks, revealed enhanced synchronization between brain regions related to reward pathways in IAD patients. A similar study of individuals with online gaming addiction (OGA) proposed using increased amplitude LFF in the left medial orbitofrontal cortex, which has anatomical connections to several regions related to goal-directed decision-making, as a biomarker for the disease [30]. Hong *et al.* used the network-based statistic (NBS) to analyze group differences in inter-regional functional connectivity between IAD and control groups, and widespread reduction of functional connectivity was observed in the IAD group with, notably, no global disruption of overall network topology [26]. In another functional connectivity-based study, alterations in default network connectivity were explored using the posterior cingulate cortex (PCC) as a seed region [24]. Results showed increased functional connectivity between the bilateral cerebellum posterior lobe and middle temporal gyrus, as well as decreased connectivity between the bilateral inferior parietal lobule and right inferior temporal gyrus.

In current study, we apply graph-theoretic approach to analyze IAD based on R-fMRI data. We first evaluate the significance of the functional connectivity disruption using **parametric tests** with multiple comparison correction. This enables us to fully explore the **full pattern of brains functional connections** and the **patterns of connectivity between large-scale networks** [31]. Second, we investigate possible connectivity disruptions associated with IAD in terms of **global network properties**, including small-worldness properties (i.e., clustering coefficient and characteristic path length) and network efficiency (i.e., global and local efficiencies) over a small-world regime. Third, with the same network sparsity range, we assess the functional importance of a network by taking into account a region's relationship with the entire functional connectome [32] based on the centrality measures of each ROI. We are motivated to use network centrality to **better localize** the disrupted regions on a more local level. Finally, we explore **relationships between network metrics and both behavioral and clinical scores** of participants. Investigating the connection between network properties and clinical outcome enhances our knowledge of addiction pathology and provides vital insight for the development of more reliable IAD diagnosis techniques.

## Materials and Methods

### Participants

Thirty-three right-handed participants, comprising 17 adolescents with IAD (15 men and 2 women) and 16 sex-, age-, and education-matched healthy control (HC) subjects (14 men and 2 women), participated in this study. The patients were recruited from the Department of Child and Adolescent Psychiatry, Shanghai Mental Health Center, School of Medicine of Shanghai Jiao Tong University. The control subjects were recruited from the local community using advertisements. The study was approved by the Medical Research Ethics Committee and Institutional Review Board of Shanghai Mental Health Center in accordance with the Declaration of Helsinki, and full written informed consent was obtained from the parents/guardians of each participant.

The duration of IAD was estimated via a retrospective diagnosis. All subjects were requested to recall their life-style when they were initially addicted to the internet. To validate their internet addiction, the patients were retested according to the modified Young's Diagnostic Questionnaire (YDQ) for internet addiction criteria by Beard and Wolf [33], and the reliability of the self-reported IAD was confirmed through interview with their parents. The IAD patients spent at least 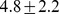 hours per day on internet or online gaming, and 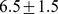 days per week. We verified this information from the roommates and classmates of the patients that they often insisted being on the internet late at night, disrupting others' lives despite the consequences. Note all the patients were addicted to internet at least or more than 2 years. Details of the modified YDQ for internet addiction criteria are provided in File S1.

Following previous IAD research [34], only those HCs who spent less than 2 hours (hour spent = 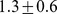) per day on the internet were included in the current study. The HC group spent 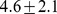 days per week on the internet. The HCs were also tested with the modified YDQ criteria to ensure they were not suffering from IAD. All recruited participants were native Chinese speakers and had never used illegal substances. Note the modified YDQ was translated to Chinese for the convenience of the participants. To further justify the diagnosis results, another IAD diagnostic measure, Young's Internet Addiction Scale (YIAS) [35], was conducted for each participant. The YIAS is a 20-item questionnaire developed by Dr. Kimberly Young to assess the degree of internet addiction. It categorizes internet users into three degrees of severity based on a 100-point score scheme: mild online user (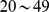 points), moderate online user (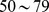 points), and severe online user (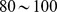 points).

Besides diagnosis of IAD via the modified YDQ and YIAS, the behavioral conditions of IAD patients were also assessed using several behavior-related questionnaires: Barratt Impulsiveness Scale-11 (BIS-11) [36], Time Management Disposition Scale (TMDS) [37], Strengths and Difficulties Questionnaire (SDQ) [38], and McMaster Family Assessment Device (FAD) [39]. Both the child and parent versions of SDQ were used in the study. Details of these questionnaires are provided in the File S1.

Before being interviewed for medical history, all participants underwent a simple physical examination (blood pressure and heartbeat tests) to exclude physical disorders related to the motion, digestive, nervous, respiratory, circulation, endocrine, urinary, and reproductive systems. The exclusionary criteria included: 1) a history of comorbid psychiatric and non-psychiatric disorders, such as anxiety disorder, depression, compulsivity, schizophrenia, autism, or bipolar disorder; 2) a history of substance abuse or dependency; 3) a history of physical disorders related to the motion, digestive, nervous, respiratory, circulation, endocrine, urinary, and reproductive systems; and 4) pregnancy or menstrual period in women during the day of scanning. This exclusionary procedure is important to ensure the participants in this study are not affected by other physical, neurological or neuropsychiatric disorders and hence reduces possible biases in the findings obtained. Detailed demographic information and clinical scores are provided in Table 1.

**Table 1:** Demographic information of the participants involved in this study.

Variables	IAD	Control	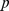 -value
No. of subjects	17	16	-
Gender	15M/2F	14M/2F	0.9484^a^
Age (years)	17.3 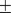 2.6	17.7 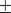 2.5	0.6297^b^
Education (years)	10.8 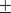 2.6	11.6 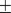 2.8	0.4029^b^
Hours of internet used (/day)	4.8 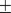 2.2	1.3 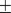 0.6	<0.001^b^
Days of internet used (/week)	6.5 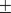 1.5	4.7 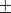 2.2	0.001^b^
YIAS	62.4 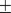 17.1	37.0 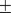 10.6	<0.001^b^
BIS-11	69.2 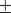 12.7	66.8 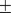 7.8	0.5154^b^
TMDS	126.5 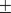 23.2	124.4 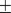 19.8	0.7890^b^
SDQ-P	21.4 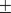 3.7	16.5 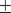 3.8	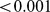 b
SDQ-C	37.3 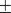 10.7	23.9 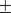 6.0	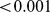 b
FAD	151.4 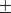 20.1	137.5 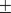 12.3	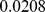 b

(YIAS = Young's Internet Addiction Scale, BIS-11 = Barratt Impulsiveness Scale-11, TMDS = Time Management Disposition Scale, SDQ-P = Strengths and Difficulties Questionnaire parent version, SDQ-C = Strengths and Difficulties Questionnaire children version, FAD = McMaster Family Assessment Device).

a The 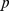 value was obtained by two-tailed Pearson Chi-square (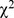) test.

b The 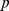 value was obtained by two-sample two-tailed 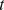-test.

### Data Acquisition and Preprocessing

Data acquisition was performed using a 3.0 Tesla scanner (Philips Achieva). Resting-state functional images of each participant were acquired with echo time (TE) = 30 ms and repetition time (TR) = 2000 ms. The acquisition matrix was 64×64 with a rectangular FOV of 230×230 mm^2^, and voxel resolution of 3.59×3.59×4 mm^3^. The scan included 220 volumes for each participant. During the data acquisition, participants were asked to lie quietly in the scanner with their eyes closed. Although no extra technique or device was used to measure whether the subjects actually kept their eyes closed, the subjects have confirmed that they were aware and kept their eyes closed during the scan.

Data preprocessing was carried out using a standard pipeline in two R-fMRI processing toolboxes, DPARSF [40] and REST [41]. Prior to any preprocessing, the first 10 R-fMRI volumes of each subject were discarded to achieve magnetization equilibrium. R-fMRI volumes were normalized to the MNI space with resolution 3×3×3 mm^3^. Regression of nuisance signals including ventricle, white matter, and global signals was performed. None of the participants were excluded based on the criterion of a displacement of more than 3 mm or an angular rotation of greater than 3 degrees in any direction. To further minimize the effects of head motion, we used Friston 24-parameter correction as well as voxel-specific mean framewise displacement (FD) [42] with FD threshold of 0.5. Prior to functional connectivity estimation, the mean R-fMRI time series of each ROI was band-pass filtered (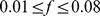 Hz).

### Network Construction and Individual Connections Analysis

Graph theoretical analysis was adopted in this study to investigate functional alterations of the brain connectome caused by IAD among a group of Chinese adolescents. Functional brain networks were constructed at a macroscale level where nodes represent the predefined brain regions and edges represent interregional resting-state functional connectivity (RSFC). To define network nodes, we parcellated the brain into 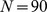 regions-of-interest (ROIs) by warping the fMRI images to the Automated Anatomical Labeling (AAL) atlas [43]. Regions based on the AAL atlas are listed in Table S1 in File S1. The representative time series of each ROI was then obtained by averaging the regressed time series over all voxels in each individual ROI. To measure interregional RSFC, we calculated the pairwise Pearson correlation for all possible ((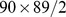) = 4005) ROI pairs and constructed a symmetric connectivity matrix to represent these connections. We analyzed group-level differences between every pair of ROIs in terms of connection strength. Significant differences for each functional connection were assessed using mass univariate (two-tailed) 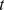-tests with a threshold of 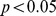 and false discovery rate (FDR) correction.

### Network Metrics and Characteristics Analysis

The Pearson correlation-based functional connectivity matrix is densely connected, with many spurious, low-strength elements. To better model human brain networks, which exhibit small-world properties, each individual's functional connectivity matrix was further processed to have a sparsity range that falls within the small-world regime (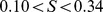) [44]–[48]. This regime ensures relatively consistent small-world characteristics for brain networks of 90 ROIs [44]. Specifically, the Pearson correlation matrix of every subject was converted into binarized adjacency matrices, 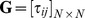, according to the predefined sparsity, where all 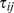 are initially set to one, and then the elements corresponding to the lowest correlation values are repeatedly set to zero until a certain level of sparsity is achieved. Based on these networks, we employed both global and regional network metrics to analyze overall architecture and regional nodal centrality of the brain networks for group-level comparison. The global metrics employed included small-world parameters, namely the clustering coefficient (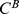) and characteristic path length (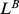) [49], [50], as well as the global network efficiency (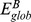) and local network efficiency (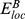). In addition, we calculated normalized versions of these measures using random networks (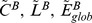, and 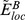) to ensure small-world property of the constructed brain networks. We define a network as small-world if it meets the following three criteria: 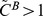, 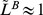, and small-world ratio, 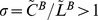. Three nodal centrality metrics – degree (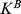), efficiency (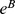), and betweenness (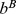) – of each brain region were calculated to investigate the local characteristics of the functional network 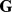 [44], [46].

To statistically investigate between-group differences, we performed two-tailed, two-sample 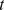-tests with a threshold of 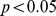 (FDR corrected) on each network metric (global and regional) based on the area under curve (AUC) of each network metric constructed from the small-world regime [48]. AUC provides a summary of the topological characteristics of brain networks over the entire small-world regime, instead of only considering the topology at a single sparsity threshold [44], [51]. Specifically, for each network metric, we first calculated the AUC value of each individual subject across networks with different levels of sparsity and then performed two-sample 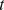-tests to statistically quantify any group-level difference between IAD and healthy groups. It is noteworthy that before the statistical tests, we applied multiple linear regressions to remove the effects of age, gender and education, as well as their interactions [31], [52]–[54].

### Reliability and Repeatability using Functional Atlas

In the current study, functional connectivity networks were constructed at a regional level by parcellating the whole brain into 90 ROIs based on the AAL atlas. However, it has also been reported that brain networks derived from different parcellation schemes or using different spatial scales may exhibit distinct topological architectures [55]–[57]. To evaluate the reliability and repeatability of our results, we repeated the experiments using the Dosenbach's functional atlas [58], which partitions the human brain into 160 ROIs, including the cerebellum. In this atlas, each ROI is defined as a 10 mm diameter square surrounding a selected seed point, and the distance between all ROI centers is at least 10 mm with no spatial overlap, meaning some brain areas are not covered by the set of ROIs.

### Relationships Between Network Metrics and Behavioral Scores

For those regions (based on the AAL atlas) that show significant group-level differences in regional nodal centrality, we used pairwise Pearson correlation (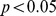, FDR corrected) to analyze the relationships between each region's network properties and an individual's behavioral scores. Specifically, in the correlation analysis, network metrics were treated as the dependent variables, while behavioral scores, i.e., BIS-11, TMDS, SDQ, and FAD, were treated as the independent variables. To further understand the relationship between the affected brain regions and disease severity, we also computed the Pearson correlation coefficient between network features and YIAS scores.

## Results

### Demographic and Clinical Characteristics

**Figure 1:**
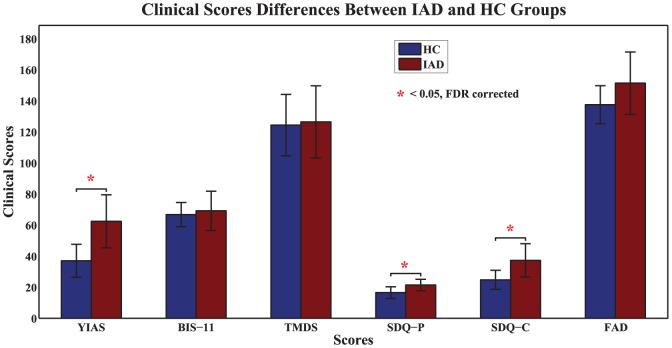
Between-group differences in terms of clinical and behavioral measures. (YIAS = Young's Internet Addiction Scale, BIS-11 = Barratt Impulsiveness Scale-11, TMDS = Time Management Disposition Scale, SDQ-P = Strengths and Difficulties Questionnaire parent version, SDQ-C = Strengths and Difficulties Questionnaire children version, FAD = McMaster Family Assessment Device).

There is no significant difference in terms of age, gender, and years of education (all with 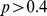) between the IAD and HC groups. However, there are significant differences in internet use in terms of days per week (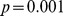) and hours per day (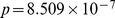). While there is no significant difference between groups for the BIS-11 and TMDS scores (all with 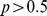), the SDQ-P (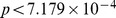), SDQ-C (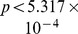), and FAD (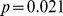) scores are significantly higher in the IAD group, as shown in Table 1 and Figure 1. Notably, the YIAS (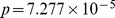), the clinical measure used to classify IAD, shows the most significant group-level difference.

### Individual Functional Connectivity

**Figure 2:**
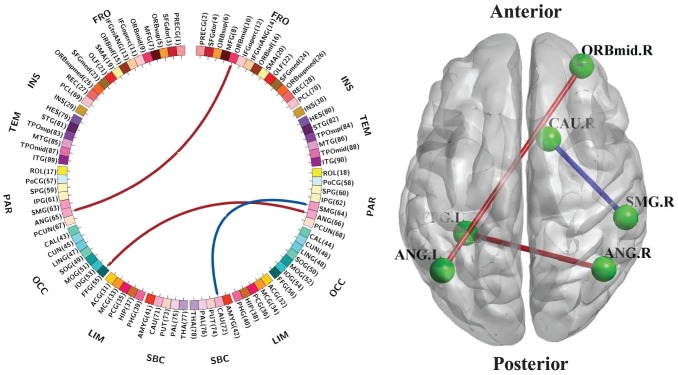
Significantly altered functional connections in IAD patients (FDR corrected). Red: increased functional connectivity, Blue: decreased functional connectivity. (FRO: Frontal, INS: Insula, TEM: Temporal, PAR: Parietal, OCC: Occipital, LIM: Limbic, SBC: Subcortical). This visualization is created using the BrainNet Viewer package (http://www.nitrc.org/projects/bnv) and the Circos (http://circos.ca/).

Compared to the HC group, only three functional connections experienced significant alteration after FDR correction. Two inter-hemispheric connections, one between the left angular gyrus (parietal lobe) and right middle orbitofrontal cortex (frontal lobe) and another between the left fusiform gyrus (occipital lobe) and right angular gyrus (parietal lobe), exhibit increased connectivity strength in IAD patients. One intra-hemispheric connection, between the right caudate (subcortical cortex) and right supramarginal gyrus (parietal lobe), shows decreased connectivity in the disease group. These significantly altered functional connections are illustrated in Figure 2. Red and blue color connections denote the increased and decreased functional connectivities, respectively, in the IAD group. Note that most of the affected functional connections involve regions located in the right hemisphere and parietal lobe.

### Global characteristics of the Functional Networks

We explored the topological properties of intrinsic functional brain networks by comparing their small-world behaviors with comparable random networks over multiple network sparsity levels, 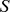. In particular, we investigated small-world parameters (e.g., clustering coefficient, characteristic path length, and small-world ratio, 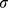), as well as the global and local efficiencies. Random networks used in the study preserved the number of nodes and edges, as well as the degree distributions of real brain networks in concern through the rewiring technique described in [59]. Statistical analyses using two-sample 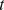-tests (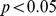, FDR corrected) on AUC values over the small-world regime demonstrated no significant difference between the IAD and HC groups in terms of global network properties.

### Regional Nodal Characteristics of Functional Networks

Despite the common small-world topology, there were significant group-level differences observed in the regional nodal centrality. In this study, we consider a brain region to be significantly altered in IAD group if at least one of its three regional nodal metrics has a 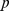-value smaller than 0.05 (FDR corrected) based on its AUC values. Table 2 summarizes the regions that are significantly altered in IAD patients. Compared to the HC group, IAD patients showed nodal centrality alterations predominantly located in the left inferior parietal lobule (IPL), left thalamus (THA), and other regions such as the limbic system, specifically the right anterior cingulate gyrus (ACG) and right middle cingulate gyrus (MCG). Notably, the IPL and ACG are components of the default-mode network (DMN), which has previously been linked to altered connectivity in substance addiction [60]–[62].

**Table 2:** Regions showing abnormal nodal centralities in the IAD patients compared with healthy controls (HC) based on the AAL atlas.

	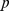 -value		
Regions	Degree	Efficiency	Betweenness
Right anterior cingulate gyrus (limbic)	0.2092	0.4844	**0.0080**
Right middle cingulate gyrus (limbic)	0.8702	0.9456	**0.0333**
Left inferior parietal lobule (parietal)	**0.0350**	0.2822	0.8362
Left thalamus (subcortical)	0.4803	0.9647	**0.0069**

Regions with 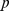-values smaller than 0.05 after multiple comparison correction using FDR (shown in bold font) in at least one of the three regional nodal centralities are considered abnormal.

### Reliability and Repeatability using Functional Atlas

When the Dosenbach's atlas is used to define ROIs, significant group differences are observed mainly in frontal and parietal connections to the cerebellem. These findings are summarized in Table 3. Although these connections differ from those identified based on the AAL atlas, most disrupted connections involve the same lobes of the brain, except for the cerebellum regions. In terms of global network metrics, we found no difference between IAD and HC groups, similar to the results based on the AAL atlas. For local network metrics, we found that some of the identified regions are located spatially near to the regions identified based on the AAL atlas, such as the ACG and THA as given in Table 4.

**Table 3:** Functional connections in the IAD individuals that experienced significant alterations based on the Dosenbach atlas.

Connections								
Region 1	x	y	z	Region 2	x	y	z	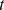 -scores
vlPFC (frontal)	46	39	−15	frontal (frontal)	58	11	14	3.9735
vent aPFC (frontal)	−43	47	2	med cerebellum (cerebellum)	5	−75	−11	4.2945
precuneus (parietal)	8	−40	50	temporal (temporal)	−53	−37	13	3.8254
dFC (frontal)	60	8	34	vFC (frontal)	43	1	12	4.4190
SMA (parietal)	0	−1	52	med cerebellum (cerebellum)	5	−75	−11	3.9768
SMA (parietal)	0	−1	52	inf cerebellum (cerebellum)	−6	−79	−33	4.1472
temporal (cerebellum)	59	−13	8	inf cerebellum (cerebellum)	32	−61	−31	3.8373
temporal (temporal)	59	−13	8	med cerebellum (cerebellum)	5	−75	−11	4.4282
mPFC (frontal)	0	51	32	med cerebellum (cerebellum)	−16	−64	−21	−3.9785
post cingulate (limbic)	−8	−41	3	post cingulate (limbic)	−5	−43	25	−3.9979

**Table 4:** Regions showing abnormal nodal centralities in IAD patients compared with healthy controls (HC) based on the Dosenbach's atlas.

				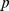 -value		
Regions	x	y	z	Degree	Efficiency	Betweenness
temporal (temporal)	51	−30	5	**0.0090**	**0.0084**	0.1412
precentral gyrus (frontal)	54	−22	22	**0.0010**	**0.0038**	0.7138
ACC (limbic)	9	39	20	0.0350	0.2822	**0.0036**
thalamus (subcortical)	−12	−3	13	0.4803	0.9647	**0.0009**

Regions with 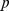-values smaller than 0.05 after multiple comparison correction using FDR (shown in bold font) in at least one of the three regional nodal centralities are considered abnormal.

### Relationships Between Network Metrics and Behavioral Measures

**Figure 3:**
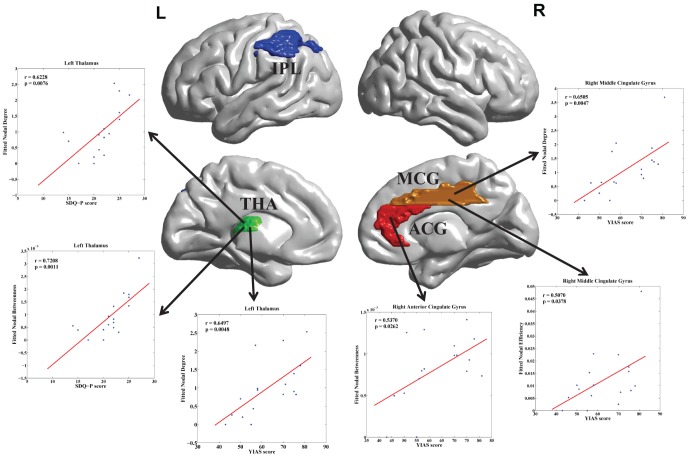
The brain regions that are significantly correlated with behavioral and clinical scores in the IAD group (FDR corrected). This illustration was created using the BrainNet Viewer package (http://www.nitrc.org/projects/bnv). (YIAS = Young's Internet Addiction Score, BIS-11 = Barratt Impulsiveness Scale-11, TMDS = Time Management Disposition Scale, SDQ-P = Strengths and Difficulties Questionnaire parent version, SDQ-C = Strengths and Difficulties Questionnaire children version.).

There is no significant (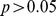, FDR corrected) correlation between global network metrics (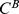, 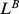, 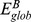, and 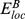) and behavioral and clinical scores. However, regional nodal metrics of several regions are significantly (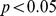, FDR corrected) correlated with behavioral and clinical scores. The right ACG is positively correlated with the YIAS score. The right MCG is positively correlated with the YIAS score. The left THA is positively correlated with the YIAS and SDQ-P scores. However, the left IPL is not significantly correlated to any behavioral or clinical score. The brain regions that are significantly correlated with the behavioral and clinical scores are shown in Figure 3.

## Discussion

### Alterations of Individual Functional Connectivity

Insights into the mechanism of human brain development is important for better understanding of the pathological underpinnings of disorders affecting children and adolescents, leading to possible early treatment. Based on the graph theoretical analysis of R-fMRI data, it has been suggested that functional organization of the human brain matures and evolves from childhood to adolescence to adulthood by following a unique trend - greater functional segregation in children and greater functional integration in adults at the whole-brain level [63]–[66]. In particular, the organization of functional brain networks shifts from local connectivity to a more distributed architecture with development [63], [66], where adults tend to have weaker short-range functional connectivity and stronger long-range functional connectivity than children [65].

Our findings demonstrate that the disrupted connections observed in IAD, although only a handful after FDR correction, are long-range and inter-hemispheric functional connections that are important for long distance communication in the human brain. The disruption of long-range and inter-hemispheric connections is a common symptom in many behavioral abnormalities, including autism [67]–[70], schizophrenia [71], opioid addiction [72], [73], and cocaine addiction [74]. Impairment of long-range connections can be seen as a failure of the integration process within a distributed functional network of the human brain [63], [64], [75], a deviation from the normal neurodevelopmental trajectory. Hence, we speculate that the abnormal development of long-range and inter-hemispheric connectivity in IAD adolescents observed in this study is one of the possible reasons for their addictive behavior.

### Alterations in Global Network Properties

The human brain is regarded as a complex and large interconnected dynamic system with various important topological properties, such as small-worldness, high efficiency at low wiring cost, and highly connected hubs [46], [76]–[79]. In a small-world network, nodes are locally clustered in favor of modular information processing and are remotely connected through a small number of long-range connections for efficient overall routing [50]. Both the IAD and HC groups demonstrated small-world properties, i.e., high clustering coefficients (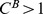) and similar characteristic path lengths (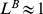), when compared with comparable random networks. However, we observed consistently larger normalized clustering coefficients and similar normalized characteristic path length in IAD group compared with HC group over the connection density, in line with previous R-fMRI studies [26]. Larger clustering coefficient reflects disrupted neuronal integration between distant regions, which show relatively sparse long-distant and relatively dense short-distant functional connections in IAD and HC groups. Progression of clinical stages, from mild to severe, may cause more impairment or disconnection of long-distant connections, and thus possibly encourage the establishment of short-distant connections within cluster as alternative paths to preserve information transmission between two distant regions. However, establishment of short-distant connections may introduce abnormal clusters that increases the risk of generating an uncontrolled or random flow of information through the entire network. On the other hand, all brain networks demonstrated similar parallel information processing of global and local efficiencies compared to comparable random network [80]. These findings support the concept of a small-world model of the human brain that provides a balanced combination of local specialization and global integration [81]. Our observation of no significant difference between IAD and HC groups in terms of global network properties may imply that the changes of functional network structure in IAD are subtle. Consequently, further research into region-specific IAD biomarkers could reveal significant information about the pathology of the disease, and of addiction, in general.

### Regional Nodal Characteristics of Functional Networks

The IAD-related alterations of nodal centrality are mainly found in limbic system components including ACG and MCG, IPL, and THA. Disturbances of these regions as well as their related connection pathways can be interpreted to reflect decreased information processing efficiency, possibly mirroring functional disruptions in IAD.

The cingulate gyrus (CG), an integral part of the limbic system, is involved in emotion formation and processing, learning and memory, executive function, and respiratory control [82]. It receives inputs from the THA and the neocortex and projects to the entorhinal cortex via the cingulum. This pathway focuses on emotionally significant events and regulates aggressive behaviors [29]. Disruption of functions related to the CG could impair an individual's ability to monitor and control his or her behaviors, especially behaviors related to emotion [83]. Most substance and behavioral addiction analyses have shown significant alterations in anterior and posterior parts of the CG (ACG and PCG), including alcohol addiction [84], pathological gambling [85], and IAD [27], [29]. In cocaine abusers, similar, additional alterations in the MCG have also been reported [86]. In previous fMRI studies, it has also been shown that the anterior, middle, and posterior CG are all affected in reward and punishment conditions [87]. Due to the role of the MCG in processing positive and negative emotions, it is not surprising that the region shows significant connectivity disruption in IAD patients.

The THA is a switchboard of brain information and is involved in many brain functions including reward processing [88], goal-directed behaviors, and cognitive and motor functions [89]. It relays sensory and motor signals from subcortical regions to the cerebral cortex [90]. Through the THA, the orbitofrontal cortex receives direct and indirect projections from other limbic brain regions that are involved with drug reinforcement, such as the amygdala, CG, and hippocampus [91], to control and correct reward- and punishment-related behaviors [92]. Abnormal thalamo-cortical circuitry found in online game addicts [93] may suggest an impairment of THA functioning related to chronic patterns of poor sleep quality [94] and overwhelming attentional focus on computer. In addition, the THA is functionally connected to the hippocampus [95] as part of the extended hippocampal system, which is crucial for cognitive functions such as spatial navigation and the consolidation of information from short-term memory to long-term memory [96], [97].

We observed significant alterations of nodal centralities in the IPL, in line with the results reported in recent R-fMRI-based IAD studies [24], [93]. Similar to the THA, the IPL is massively connected to the auditory, visual, and somatosensory cortexes, and it is able to process different kinds of stimuli simultaneously. As one of the last developed structures of the human brain in the course of development, the IPL may be more vulnerable to the excessive exposure of auditory and visual stimuli, particularly during childhood. IPL impairment induced by internet overuse may suppress the ability of an individual to properly mediate response inhibition of impulse regulation [98], [99], damaging their ability to resist cue-induced internet cravings, which may further impair the IPL. Such circular patterns are often seen in substance and behavioral addicts.

Regions of the DMN are commonly more active at rest than performing goal-directed tasks [62]. These regions known to be involved in emotional modulation and self-referential activities, including evaluating salience of internal and external cues, remembering the past, and planning the future [60], [62], which are the important criteria in diagnosis IAD. It has previously been suggested that altered connectivity involving the DMN regions contributes to various symptomatic behaviors in diseases [100], including substance addictions [101], [102] and behavioral addictions [24], [103]. Our findings of altered of functional connectivity involving several regions of DMN is partially consistent with the previous observations, which suggests the DMN has the potential to serve as a biomarker for identifying IAD patients.

### Reliability and Repeatability using Functional Atlas

Some of the abnormal brain regions identified based on the AAL atlas were also identified using the functional atlas, supporting the reliability and repeatability of our results. One possible reason of the slightly different results is the regime of 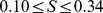 used in this study. The small-world characteristics of connectivity networks constructed based on the AAL atlas of 90 ROIs is most consistent within this range [44]. However, this sparsity range may not be optimal for atlases with different numbers of ROIs. Furthermore, ROIs obtained from the Dosenbach atlas are defined functionally and do not cover the whole brain [58]. In this atlas, centers of all 160 ROIs are first identified and a sphere with a radius of 5 mm is grown from each center, producing a 10 mm spherical ROI. The center of each ROI is also set to be at least 10 mm apart from the centers of other ROIs, leading to spatially non-overlapping atlas. On the other hand, the AAL atlas covers the gray matter tissue of the whole cerebrum. These differences in ROI definition and overall area covered may contribute to the variations of the results. Hence, further research using a larger cohort is necessary to determine the extent the choice of brain parcellation scheme affects the characterization of network topology.

### Correlation Between Network Metrics and Behavioral Measures

In this study, we did not observe any correlation between global network metrics and behavioral measures, implying the absence of alterations in whole brain network topology. This finding may also suggest that the variations of brain network is subtle due to the plasticity of the human brain (neuroplasticity) [104], [105] in recovering most of its daily functions via alternative pathways (neural circuitry). Brain plasticity involves reorganization of connections between nerve cells or neurons and can be influenced by a myriad of factors [106]–[108]. It happens in an age-related manner with greater prevalent during childhood and adolescence than adulthood, suggesting a better recovery of impaired neuronal connections in adolescents with IAD. Furthermore, it has been shown that a variety of behavioral conditions, ranging from addiction to neurological and psychiatric disorders, are correlated with localized changes in neural circuits [106]. It is thus not surprising that coarse level global network measures such as mean clustering coefficient, characteristic path length, and network efficiencies are less sensitive in detecting brain circuitry changes in the IAD group.

However, regional nodal metrics of several brain regions are correlated with some of the behavioral measures. In particular, the parent version of SDQ (SDQ-P), which measures both the ability of an individual to appropriately handle impulsiveness and the severity of emotion and prosocial behavior problems based on the information provided by the parents of the studied adolescents, is positively correlated with the functionally affected brain regions found in IAD. The inability to control impulsive behaviors and emotions is one of the main behavioral symptoms. It is common that the patients do not aware of the changes to their emotions and behaviors although these changes are relatively obvious to people surrounding them. This may be the main reason why none of the network measures are correlated with the children version of SDQ (SDQ-C) due to its self-assessment nature. On the other hand, there is no significant correlation between regional network measures and other behavioral measures including BIS-11, FAD, and TMDS. This finding is supported by the large 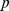-values for these measures between the IAD and healthy groups (Table 1). These findings may suggest that some of these behavioral measures are useful to determine affected regions and hence help IAD diagnosis, although a significant amount of work is still required to better understand the roles of these measures in behavioral addictions or disorders.

### Methodological Issues/Limitations

There are several limitations that should be highlighted in this study. First, the diagnosis of IAD was mainly based on results from self-reported questionnaires, which might affect the reliability of diagnoses. In the future, standardized diagnostic tools for IAD identification must be developed to improve the reliability and validity of IAD diagnoses. Second, our study is limited by the small sample size and the imbalance of the gender of the participants (31 males and 4 females), which might reduce the statistical power and generalizability of the findings, although these factors have been controlled in analysis. The effect of gender on IAD prevalence is still a debated issue. Based on the findings of Young [35], a high number of females exhibit internet dependence. In contrast, one recent study reported that males display a higher risk of IAD behavior [109]. However, it has also been reported that there is no relationship between gender and IAD [110], [111]. Future experiments using a larger cohort with a more balanced gender ratio are required to better assess the relationship between gender and IAD susceptibility.

## Supporting Information

File S1 **Supplementary Materials.**
(PDF)
