# Supplementary material for: Disrupted Brain Functional Network in Internet Addiction Disorder: A Resting-State Functional Magnetic Resonance Imaging Study
Source: PLoS One. 2014 Sep 16;9(9):e107306. doi: 10.1371/journal.pone.0107306 (PMC4165900; doi:10.1371/journal.pone.0107306)
Supplement: File S1 — Supplementary Materials. (PDF) [file pone.0107306.s001.pdf]

Supplementary Materials

Young's Diagnostic Questionnaire (YDQ) for Internet Addiction

Young's Diagnostic Questionnaire (YDQ) for internet addiction was adapted from the DSM-IV [1] based on criteria for pathological gambling. According to the prescription provided by the YDQ, a person is considered having IAD if he or she meets at least five or more of following criteria [2]:

1. Preoccupation: Thoughts about previous on-line activity or anticipation of the next on-line session.
2. Tolerance: Needs for increasing amounts of time on the internet to achieve satisfaction.
3. Repeated, unsuccessful efforts to control, reduce or stop internet use.
4. Withdrawal symptoms, such as anger, depression, mood swings, anxiety, fear, irritability, sadness, loneliness, boredom, restlessness and procrastination, are developed within days to months after reduction or cessation of internet use.
5. Access internet longer than originally intended.
6. Jeopardized or risked loss of significant relationships, job, educational or career opportunities because of internet use.
7. Lies to family members, therapists, or others to conceal the extent of involvement with the internet.
8. Use of the internet is a way to escape from problems or to relieve a dysphoric mood such as feelings of hopelessness, guilty, anxiety, depression, or stress.

Young asserted that five or more "yes" responses to the eight questions mentioned above indicate a dependent user. However, Beard and Wolf concerned with the criteria in YDQ: 1) The objectivity and reliance on self-report since some of the original criteria in YDQ can be easily admitted or denied by the participants and thus make the accuracy of diagnosis questionable; 2) Some of the terms are vague and clarification is required to minimize ambiguity; and 3) It is questionable that the criteria of pathological gambling is the most accurate basis to be used for IAD diagnosis. Due to these concerns, a modified version of YDQ, which provides a more conservative estimation of the prevalence and a more stringent scoring procedure of internet addiction, was proposed [3]. Specifically, in this modified version respondents who answered "yes" to questions 1 through 5 and at least any one of the remaining three questions were considered as suffering from internet addiction. This is because the first five criteria can be met without any impairment in daily life while the last three criteria impact a person's ability to cope and function.

Young's Internet Addiction Scale (YIAS)

Young's Internet Addiction Scale (YIAS), which composes of 20 questions with 5 Likert scales [4], is commonly used to assess the severity of IAD or any online related activity. An individual is considered as nonaddict if having score lower than 50. Scores between 50 to 70 correspond to mild internet addiction with occasional problems of internet use, while scores larger than 80 indicate severe internet addiction.

Behavioral Assessments

Four questionnaires were used to assess the participant's behavioral conditions, namely the Barratt Impulsiveness Scale-11 (BIS-11) [5], Time Management Disposition Scale (TMDS) [6], Strengths and Difficulties Questionnaire (SDQ) [7], and McMaster Family Assessment Device (FAD) [8]. The BIS-11 is a questionnaire designed to assess the personality/behavioral construct of impulsiveness using a 30-item, 120-point score scheme. It has been widely used to understand the relationship of this construct with other clinical phenomena. The factors assessed by BIS-11 include attention, cognitive instability, motor, perseverance, self-control, and cognitive complexity. The lower the BIS-11 score, the better an individual response appropriately to impulsiveness.

The TMDS is a 44-item instrument used to evaluate time management dispositions of adolescents in terms of the sense of time value (social-oriented time value and individual-oriented time value), the sense of time control (setting goals, planning, priorities, time allocation, and feedback), and the sense of time efficacy (efficacy of time management and efficacy of time management behaviors). The higher the scores the better an individual's ability in time management. The SDQ comprises 25 items that are divided into five scales of five items each: hyperactivity, emotional symptoms, conduct problems, peer problems and prosocial behavior. Each item has three possible answers, each of which is assigned a value 0, 1 or 2. The score for each scale is generated by adding up the scores on the five items within that scale, producing scale scores ranging from 0 to 10. In the current study, we used the parent and child versions of SDQ to assess psychological morbidity. The lower the score, the better an individual's ability to control his emotion and his interactions with social. The FAD is a questionnaire designed to evaluate the healthiness of families according to the McMaster Model of Family Functioning [9]. It consisted of 53 items that are divided into seven categories: problem solving (5 items), communication (6 items), roles (8 items), affective responsiveness (6 items), affective involvement (7 items), behavior control (9 items), and general functioning (12 items). Each family member rates his or her agreement or disagreement with how well an item describes their family by selecting among four alternative responses: strongly agree, agree, disagree, and strongly disagree [8]. A family with lower score is considered healthier than family with higher score.

Table S1: Regions based on the AAL atlas [10].

Index	Region	Abbrev.	
		Left	Right
1, 2	Precentral gyrus	PreCG.L	PreCG.R
3, 4	Superior frontal gyrus (dorsal)	SFGdor.L	SFGdor.R
5, 6	Orbitofrontal cortex (superior)	ORBsupb.L	ORBsupb.R
7, 8	Middle frontal gyrus	MFG.L	MFG.R
9, 10	Orbitofrontal cortex (middle)	ORBmid.L	ORBmid.R
11, 12	Inferior frontal gyrus (opercular)	IFGoperc.L	IFGoperc.R
13, 14	Inferior frontal gyrus (triangular)	IFGtriang.L	IFGtriang.R
15, 16	Orbitofrontal cortex (inferior)	ORBinf.L	ORBinf.R
17, 18	Rolandic operculum	ROL.L	ROL.R
19, 20	Suplementary motor area	SMA.L	SMA.R
21, 22	Olfactory	OLF.L	OLF.R
23, 24	Superior frontal gyrus (media)	SFGmed.L	SFGmed.R
25, 26	Orbitofrontal cortex (medial)	ORBmed.L	ORBmed.R
27, 28	Rectus gyrus	REC.L	REC.R
29, 30	Insula	INS.L	INS.R
31, 32	Anterior cingulate gyrus	ACG.L	ACG.R
33, 34	Middle cingulate gyrus	MCG.L	MCG.R
35, 36	Posterior cingulate gyrus	PCG.L	PCG.R
37, 38	Hippocampus	HIP.L	HIP.R
39, 40	ParaHippocampal gyrus	PHG.L	PHG.R
41, 42	Amygdala	AMYG.L	AMYG.R
43, 44	Calcarine cortex	CAL.L	CAL.R
45, 46	Cuneus	CUN.L	CUN.R
47, 48	Lingual gyrus	LING.L	LING.R
49, 50	Superior occipital gyrus	SOG.L	SOG.R
51, 52	Middle occipital gyrus	MOG.L	MOG.R
‘ 53, 54	Inferior occipital gyrus	IOG.L	IOG.R
55, 56	Fusiform gyrus	FFG.L	FFG.R
57, 58	Postcentral gyrus	PoCG.L	PoCG.R
59, 60	Superior parietal gyrus	SPG.L	SPG.R
61, 62	Inferior parietal lobule	IPL.L	IPL.R
63, 64	Supramarginal gyrus	SMG.L	SMG.R
65, 66	Angular gyrus	ANG.L	ANG.R
67, 68	Precuneus	PCUN.L	PCUN.R
69, 70	Paracentral lobule	PCL.L	PCL.R
71, 72	Caudate	CAU.L	CAU.R
73, 74	Putamen	PUT.L	PUT.R
75, 76	Pallidum	PAL.L	PAL.R
77, 78	Thalamus	THA.L	THA.R
79, 80	Heshl gyrus	HES.L	HES.R
81, 82	Superior temporal gyrus	STG.L	STG.R
83, 84	Temporal pole (superior)	TPOsup.L	TPOsup.R
85, 86	Middle temporal gyrus	MTG.L	MTG.R
87, 88	Temporal pole (middle)	TPOmid.L	TPOmid.R
89, 90	Inferior temporal	ITG.L	ITG.R

References

1. American Psychiatric Association (2000) Diagnostic and Statistical Manual of Mental Disorders, Fourth Edition - Text Revision (DSMIV-TR). American Psychiatric Association.

2. Young KS (1998) Internet addiction: The emergence of a new clinical disorder. Cyberpsychol Behav 1: 237 - 244.

3. Beard KW, Wolf EM (2001) Modification in the proposed diagnostic criteria for internet addiction. Cyberpsychol Behav 4: 377 - 383.

4. Young KS (1996) Psychology of computer use: Xl. addictive use of the internet: a case that breaks the stereotype. Psychol Rep 79: 899 - 902.

5. Patton JH, Stanford MS, Barratt ES (1995) Factor structure of the barratt impulsiveness scale. J Clin Psychol 51: 768 - 774.

6. Huang X, Zhang Z (2001) The compiling of adolescence time management disposition inventory. Acta Psychol Sin 33: 338 - 343.

7. Goodman R (1997) The strengths and difficulties questionnaire: A research note. J Child Psychol Psychiatry 38: 581 - 586.

8. Epstein NB, Baldwin LM, Bishop DS (1983) The McMaster Family Assessment Device. J Marital Fam Ther 9: 171 - 180.

9. Epstein NB, Bishop DS, Levin S (1978) The McMaster Model of Family Functioning. J Marital Fam Ther 4: 19 - 31.

10. Tzourio-Mazoyer N, Landeau B, Papathanassiou D, Crivello F, Etard O, et al. (2002) Automated anatomical labeling of activations in SPM using a macroscopic anatomical parcellation of the MNI MRI single-subject brain. Neuroimage 15: 273 - 289.
